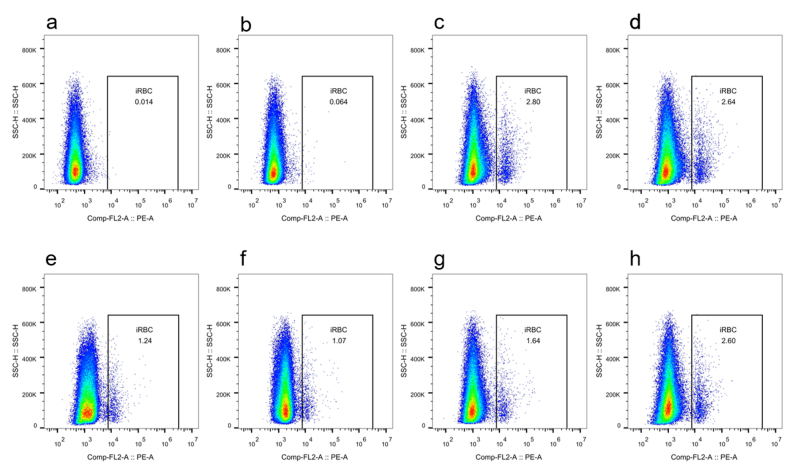# The *Plasmodium vivax* MSP1P-19 is involved in binding of reticulocytes through interactions with the membrane proteins band3 and CD71

**DOI:** 10.1016/j.jbc.2024.107285

**Published:** 2024-04-16

**Authors:** Shenghuan Zuo, Jiachen Lu, Yifan Sun, Jing Song, Su Han, Xin Feng, Eun-Taek Han, Yang Cheng

**Affiliations:** 1 Laboratory of Pathogen Infection and Immunity, Department of Clinical Medicine, Wuxi School of Medicine, Jiangnan University, Wuxi, Jiangsu, China; 2 Affiliated Hospital of Jiangnan University, Wuxi, Jiangsu, China; 3 Department of Medical Environmental Biology and Tropical Medicine, School of Medicine, Kangwon National University, Chuncheon, Gangwon-do, Republic of Korea

**Keywords:** *plasmodium*, invasion, reticulocyte, membrane protein, receptor, band3, CD71, protein–protein interaction, parasite

## Abstract

The parasite *Plasmodium vivax* preferentially invades human reticulocytes. Its merozoite surface protein 1 paralog (PvMSP1P), particularly the 19-kDa C-terminal region (PvMSP1P-19), has been shown to bind to reticulocytes, and this binding can be inhibited by antisera obtained by PvMSP1P-19 immunization. The molecular mechanism of interactions between PvMSP1P-19 and reticulocytes during *P. vivax* invasion, however, remains unclear. In this study, we analyzed the ability of MSP1P-19 to bind to different concentrations of reticulocytes and confirmed its reticulocyte preference. LC-MS analysis was used to identify two potential reticulocyte receptors, band3 and CD71, that interact with MSP1P-19. Both PvMSP1P-19 and its sister taxon *Plasmodium cynomolgi* MSP1P-19 were found to bind to the extracellular loop (loop 5) of band3, where the interaction of MSP1P-19 with band3 was chymotrypsin sensitive. Antibodies against band3-P5, CD71, and MSP1P-19 reduced the binding activity of PvMSP1P-19 and *Plasmodium cynomolgi* MSP1P-19 to reticulocytes, while MSP1P-19 proteins inhibited *Plasmodium falciparum* invasion *in vitro* in a concentration-dependent manner. To sum up, identification and characterization of the reticulocyte receptor is important for understanding the binding of reticulocytes by MSP1P-19.

With an estimated 229 million people infected and 409,000 deaths in 2019, malaria remains a major public health concern (1). The parasite *Plasmodium vivax* (*P. vivax*) is the most common *Plasmodium* species which causes malaria in humans and threatens millions of lives yearly in Southeast Asia and South America (2). Although the disease has long been regarded as a benign infection, recent research has revealed that *P. vivax* infections can also cause serious and life-threatening complications (3, 4, 5). The development of a vaccine against *P. vivax* is majorly hampered by the lack of a continuous *in vitro* culture system and an ideal animal model (6). Besides, the increasing virulence (7) and resistance of *P. vivax* to first-line antimalarial drugs necessitates the need for new antimalarial drugs and vaccines (8, 9).

The clinical symptoms and pathology of *P. vivax* infections manifest during the asexual blood stage (10), which implies that blocking the invasion of *Plasmodium* into erythrocytes can significantly alleviate malarial symptoms. It is known that *Plasmodium* invasion depends on interactions between ligands on the surface of merozoites and receptors on the surface of erythrocytes (11). The binding of the *P. vivax* Duffy binding protein (PvDBP) to erythrocyte Duffy antigen receptor for chemokines leads to a successful invasion (12). However, a study involving *P. vivax*-infected individuals in a Duffy-negative population suggested that *P. vivax* can invade erythrocytes using receptors other than the Duffy antigen (7, 13). The interactions between *P. vivax* reticulocyte-binding protein 2b and transferrin receptor 1, for example, is essential for *P. vivax* invasion of reticulocytes (14, 15). Recently, CD98 was identified as a reticulocyte membrane receptor for *P. vivax* reticulocyte-binding protein 2a and was closely associated with *P. vivax* reticulocyte preference (16).

It has been shown that the *P. vivax* merozoite surface protein 1 paralog (PvMSP1P) is a surface antigen and a novel erythrocyte-binding ligand for *P. vivax* (17). The primary structure of PvMSP1P contains a glycosylphosphatidylinositol (GPI)-anchored domain and two epidermal growth factor (EGF)-like structural domains at the C-terminal end (18, 19). This EGF-like structural domain is essential for parasite ligand binding to the erythrocyte receptor (20) and causes high levels of acquired immune responses in patients (17, 18). This makes 19-kDa C-terminal region of PvMSP1P (PvMSP1P-19) an ideal candidate for the *P. vivax* vaccine. *Plasmodium cynomolgi* (*P. cynomolgi*), a sister taxon of *P. vivax*, shares many biological and genetic similarities with *P*. *vivax*, with efforts being made in establishing a continuous *in vitro* culture system for its erythrocytic stage. Based on these facts, we studied *P. vivax* using *P. cynomolgi* as a model (21, 22).

The *P. vivax* parasite generally prefers to invade reticulocytes (23). To better understand this mechanism, we used the LC-MS analysis and identified band3 and CD71 as reticulocyte receptors for MSP1P-19. We found that antibodies against band3 loop 5, CD71, and MSP1P-19 inhibited the binding of MSP1P-1P to reticulocytes and that MSP1P-19 proteins inhibited *Plasmodium falciparum* invasion *in vitro*. These findings are crucial for elucidating the molecular mechanism of the interaction between MSP1P-19 and band3/CD71 and provide a strong base for the production of a blood-stage antimalarial vaccine.

## Results

### Expression and purification of MSP1P-19

**Figure 1:**
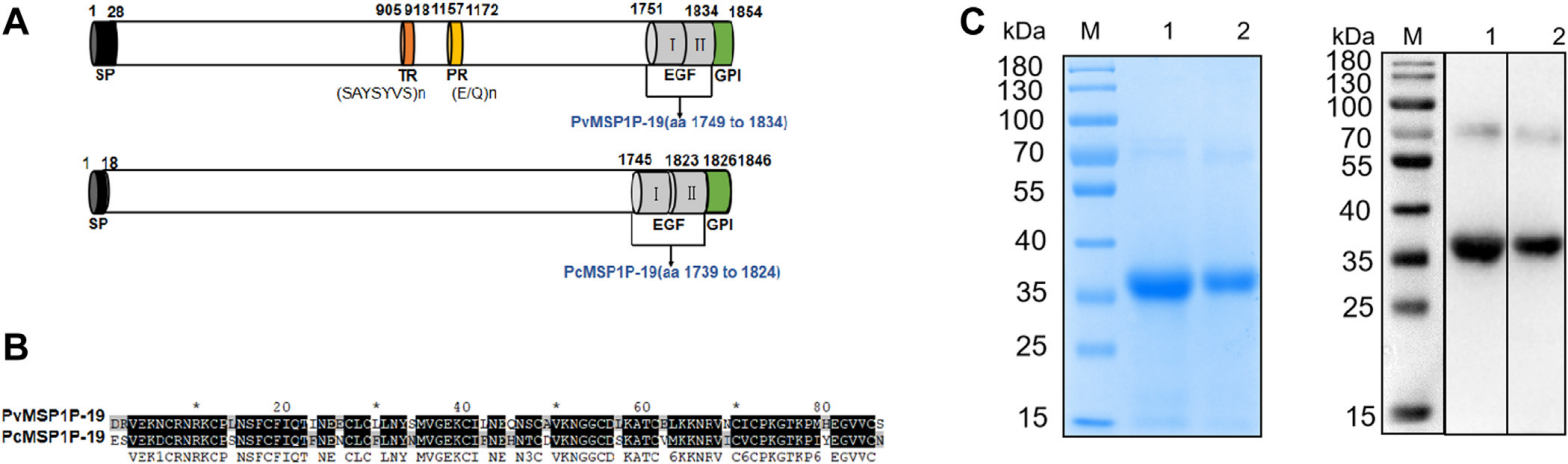
**Schematic representation of the structure and expression of recombinant PvMSP1P-19 and PcMSP1P-19 proteins.***A*, schematic diagram of MSP1P protein primary structures. The signal peptide (SP, *black*), TR domain (*orange*), PR domain (*yellow*), EGF-like domain (*gray*), and GPI anchor (*green*) are depicted in the diagram. *B*, amino acid sequence homology of PvMSP1P-19 and PcMSP1P-19. *C*, expression and purification of MSP1P proteins. The purified MSP1P-19 recombinant proteins were separated on Coomassie blue-stained SDS-PAGE gels and probed by Western blot analysis. Lanes M, molecular weight marker; Lanes 1, PvMSP1P; Lanes 2, PcMSP1P. EGF, epidermal growth factor; GPI, glycosylphosphatidylinositol; PR, polymorphic Glu- and Gln-rich; PvMSP1P, *P. vivax* merozoite surface protein 1 paralog; PvMSP1P-19, 19-kDa C-terminal region of PvMSP1P; TR, tandem repeat.

PvMSP1P is an 1854-amino acid (aa) protein and contains a signal peptide (aa 1–28), a heptapeptide tandem repeat domain (aa 905–918), a polymorphic Glu- and Gln-rich domain (aa 1157–1172), two highly conserved EGF-like domains (aa 1751–1834), and a GPI-anchored domain (aa 1834–1854). PcMSP1P is an 1846-aa protein that contains a signal peptide (aa 1–18), two EGF-like domains (aa 1745–1823), and a GPI-anchored domain (aa 1826–1846). The PvMSP1P-19 domain shows high structural similarity to PcMSP1P-19, with 79.5% aa sequence similarity (Fig. 1, *A* and *B*).

The codon-optimized *pvmsp1p-19* and *pcmsp1p-19* (Table S2), both with N-terminal Flag tags, were cloned into the pET32a vectors and expressed in *Escherichia coli* as histidine (His)-tagged proteins that fused thioredoxin (Trx). The MSP1P-19 proteins were then purified and validated by SDS-PAGE followed by Western blot analysis using an anti-His antibody, where they migrated as ∼36 kDa bands (Fig. 1*C*).

### MSP1P-19 proteins show a strong preference for the reticulocytes

**Figure 2:**
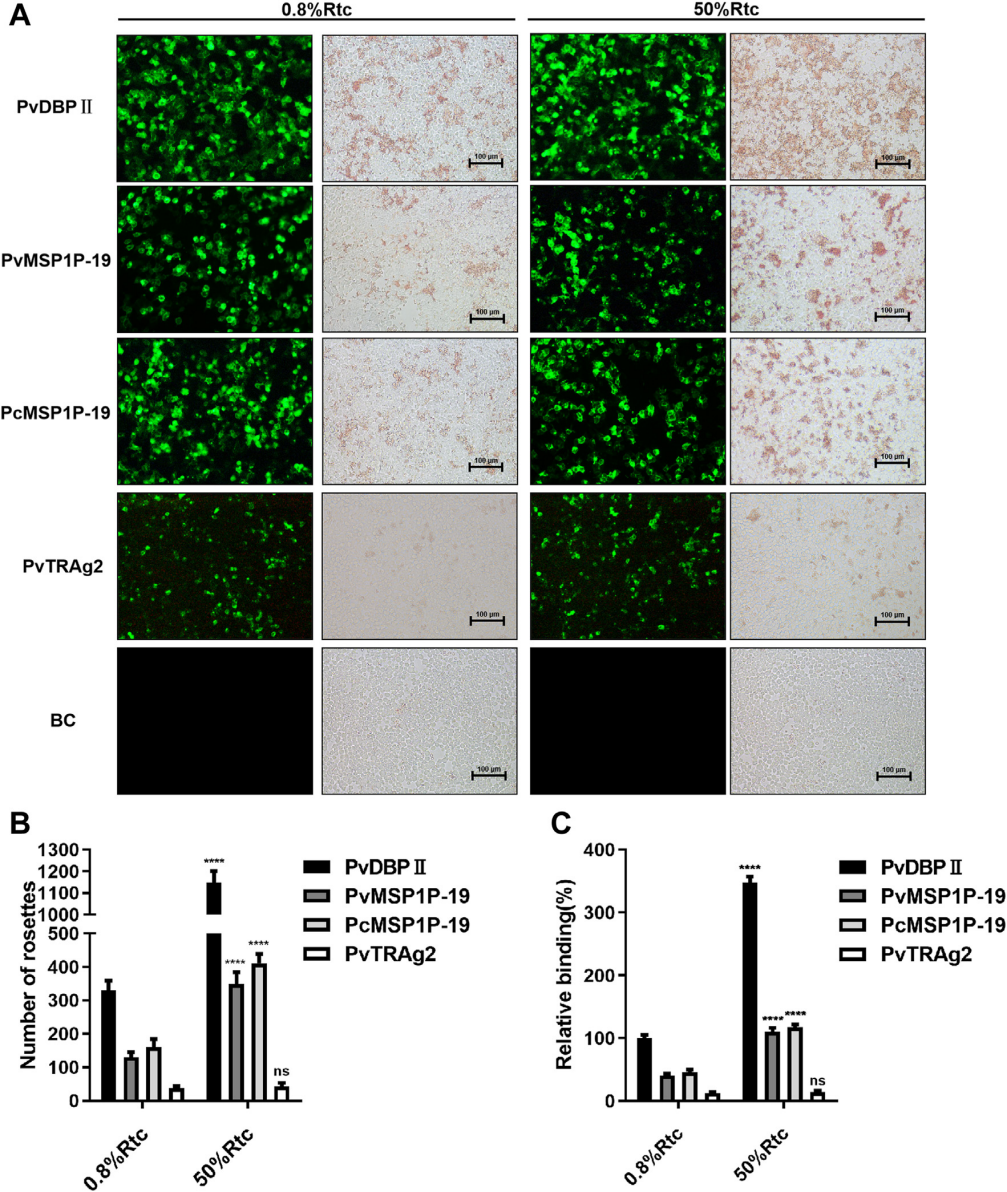
**Confirmation of MSP1P-19 preference for reticulocytes by rosetting assay.***A*, transfected or untransfected HEK293T cells were incubated with reticulocytes at different concentrations (0.8% or 50%). The rosettes and GFP-positive HEK293T cells were counted under bright field microscopy and fluorescence microscopy, respectively. Scale bars, 100 μm. *B*, quantified analysis of rosettes formed by transfected HEK293T cells incubated with different concentrations of reticulocytes. *C*, relative binding rates of transfected HEK293T cells to different reticulocyte concentrations. The relative binding capacity at different concentrations of reticulocytes was evaluated by setting the binding capacity of PvDBPII (100% transfection efficiency of HEK293T cells) with 0.8% reticulocyte as the standard (100%). Data are expressed as mean ± S.D in three independent experiments. (∗∗∗∗ *p* < 0.0001). BC, untransfected HEK293T cells; PvDBP, *P. vivax* Duffy binding protein; PvMSP1P, *P. vivax* merozoite surface protein 1 paralog; PvMSP1P-19, 19-kDa C-terminal region of PvMSP1P.

PvDBPII is well established as a protein that binds to reticulocytes. To confirm the binding of MSP1P-19 to reticulocytes, we incubated PvDBPII- (positive control), PvTRAg2- (negative control), or MSP1P-19-transfected HEK 293T cells with reticulocytes (0.8% or 50% hematocrit) and counted rosette formations under a light microscope. The transfection efficiency was assessed *via* calculating the proportion of cells expressing the green fluorescent protein (GFP) under a fluorescence microscope. PvDBPII, PvMSP1P-19, and PcMSP1P-19 were found to have transfection efficiencies of 50%, 48%, and 53%, respectively, in three independent experiments (Fig. 2*A*). The binding rate was normalized to the rosette number at 100% transfection efficiency (rosette number/transfection efficiency/total cell number), while the binding activities of PvDBPII at 100% transfection efficiency to reticulocytes (0.8% hematocrit) was set as 100% relative binding. The number of rosettes and the relative binding rates increased dramatically in the group of reticulocytes at 50% hematocrit compared to the group of reticulocytes at 0.8% hematocrit (Fig. 2, *B* and *C*).

### Identification of reticulocyte receptors for PvMSP1P-19

**Figure 3:**
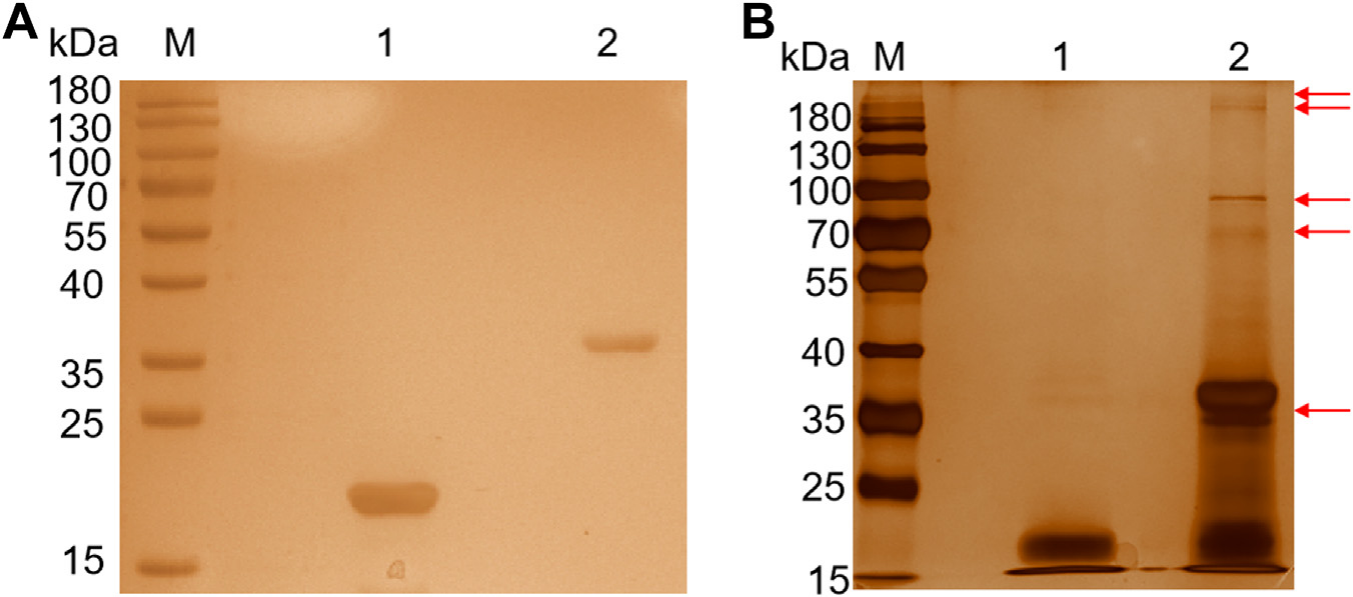
**Identification of reticulocyte receptors for PvMSP1P-19.***A*, silver-stained SDS-PAGE was used to analyze the purified proteins. Lane M, molecular mass marker. Lane 1, Trx-His-tagged protein. Lane 2, Trx-His-tagged PvMSP1P-19. *B*, the immobilized Trx-His-PvMSP1P-19 along with Trx-His tag protein were incubated with reticulocyte membrane proteins. The binding complexes were analyzed by silver-stained SDS-PAGE after washing and eluting. Lane M, molecular mass marker. Lane 1, incubation of reticulocyte membrane proteins with Trx-His-tagged protein. Lane 2, incubation of reticulocyte membrane proteins with Trx-His-tagged PvMSP1P-19. *Arrows* indicate the different protein bands. His, histidine, PvMSP1P, *P. vivax* merozoite surface protein 1 paralog; PvMSP1P-19, 19-kDa C-terminal region of PvMSP1P; Trx, thioredoxin.

To identify the reticulocyte receptors for PvMSP1P-19 binding, with Trx-His-tagged protein as a negative control, the Trx-His-tagged PvMSP1P-19 protein which was immobilized on to affinity resin was purified and incubated with reticulocyte membrane proteins. The binding compounds were separated on 10% SDS-PAGE gels and then silver stained after elution. (Fig. 3, *A* and *B*). Compared with the pure proteins group, the stained gel showed different protein bands. Six different reticulocyte membrane proteins were identified by in-gel digestion and LC-MS analyses, including band3, CD71, ANK1, solute carrier family 2, facilitated glucose transporter member 1, a 55 kDa erythrocyte membrane protein, and protein band 4.2 (Table 1). In particular, CD71 and band3 contained the maximum number of unique peptides. According to previous studies, band3 served as an essential receptor of erythrocyte for multiple *Plasmodium* invasion-related proteins (2, 24, 25). In addition, CD71, a biomarker of reticulocytes, is expressed only on reticulocytes (26, 27). Therefore, these results identified band3 and CD71 as promising reticulocyte receptors for PvMSP1P-19.

**Table 1:** Identification of reticulocyte receptors for PvMSP1P-19 by LC-MS

No.	Protein	UniPort accession no.	Coverage (%)	Molecular mass (kDa)	No. of unique peptides
1	Anion transport protein (Band3)	P48751	33.48	101.7	24
2	Transferrin receptor protein1 (CD71)	P02786	21.71	84.8	13
3	Ankyrin −1	P16157	7.6	206.1	9
4	Solute carrier family 2, facilitated glucose transporter member 1	P11166	4.33	54.1	2
5	55 kDa erythrocyte membrane protein	Q00013	5.35	52.3	2
6	Protein band 4.2	P16452	1.88	77.0	1

### Extracellular loop 5 of band3 serves as a critical binding region for MSP1P-19

**Figure 4:**
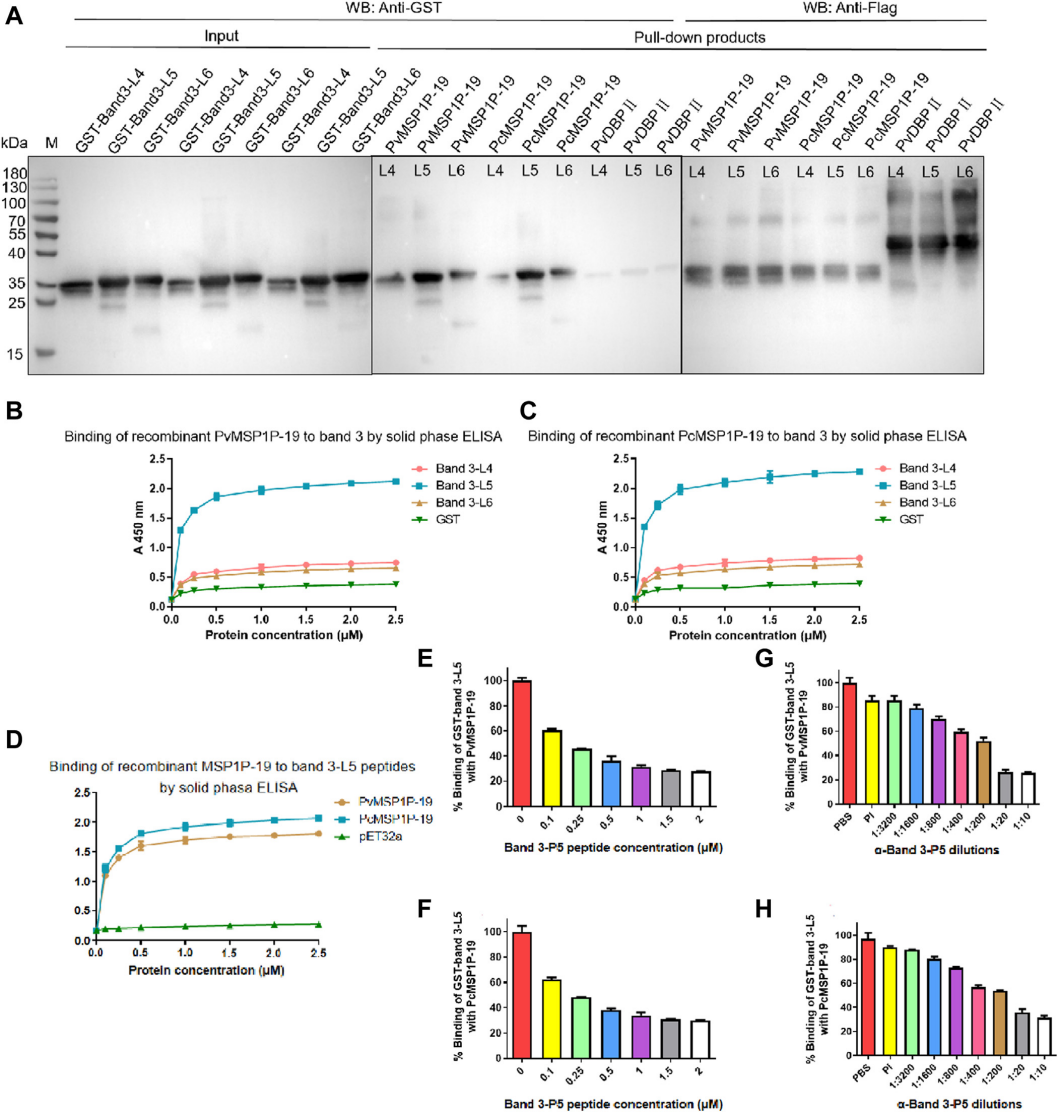
**Binding of MSP1P-19 to band3.***A*, Flag-pull-down assays. Different GST tag proteins were incubated with Flag-MSP1P-19 or PvDBPII protein, which was bound to magnetic beads, respectively. After washing and elution, the bound complexes were probed using anti-GST and anti-Flag antibodies. Results showed binding of MSP1P-19 to band3-L5 and weak binding of MSP1P-19 to band3-L4 or band3-L6. *B* and *C*, solid-phase ELISA. Different concentrations of GST-tagged proteins were incubated with PvMSP1P-19 (*B*) or PcMSP1P-19 (*C*) coated on ELISA plates. Bound complexes were detected by rabbit anti-GST antibody and goat anti-rabbit IgG antibody. *D*, different concentrations of recombinant His-MSP1P-19 or Trx-His-tagged proteins (negative control) (0–2.5 μM) were added to the ELISA plates coated with band3-P5 peptide. The bound complexes were detected using mouse anti-His monoclonal antibody. *E* and *F*, specificity of the binding of MSP1P-19 to band3-L5 determined by competition assays. Untagged band3-P5 was incubated with recombinant PvMSP1P-19 (*E*) or PcMSP1P-19 (*F*) immobilized on ELISA plate wells at increasing concentrations (0–2 μM). After washing, GST-tagged band3-L5 (2 μM) was added to the wells followed by detection with GST monoclonal antibody. Binding without the untagged band3-P5 group was used as a percentage control for the remaining concentration. The mean value is plotted with S.D in three independent experiments. *G* and *H*, antibody inhibition assays. Different dilutions of mouse anti-band3-P5 antibody were incubated with recombinant GST-tagged band3-L5 immobilized on ELISA plate wells. After washing, a fixed concentration of Flag-tagged PvMSP1P-19 (*G*) or PcMSP1P-19 (*H*) protein (2 μM) was added to the wells followed by detection using anti-Flag antibody. The mean value is plotted with S.D in three independent experiments. His, histidine; PvDBP, *P. vivax* Duffy binding protein; PvMSP1P, *P. vivax* merozoite surface protein 1 paralog; PvMSP1P-19, 19-kDa C-terminal region of PvMSP1P; Trx, thioredoxin.

According to the results of LC-MS assays (Table S1), the latter three extracellular loops of band3 (L4, L5, and L6) (28) were PCR amplified with C-terminal HA tags and cloned into the GST-pGEX-6P-1 vectors, followed by the expression of GST-HA-tagged proteins. The primers were listed in Table S4. To check the binding of band3 and MSP1P-19, we performed the Flag-pull-down assays for which band3 proteins were incubated with MSP1P-19 and PvDBPII proteins immobilized on affinity resin. As expected, the three recombinant GST-tagged proteins migrated as one 32 kDa and two 33 kDa bands, respectively (Fig. 4*A*). We observed that MSP1P-19 bound strongly to the band3-L5 but only weakly to the band3-L4 or -L6 (Fig. 4*A*). Clearly, band3-L5 was the core binding region for MSP1P-19. For ELISA assays, PvMSP1P-19 or PcMSP1P-19 protein was coated on ELISA plates and incubated with GST-tagged proteins. Results of the assay further confirmed that band3-L5 played a key role in the binding of band3 to MSP1P-19 (Fig. 4, *B* and *C*). To eliminate the effects of GST, the band3-P5 peptide corresponding to the aa sequence of the band3-L5 extracellular loop region was synthesized. With Trx-His tag protein served as the negative control, recombinant MSP1P-19 proteins were incubated with band3-P5 peptide coated on ELISA plates. Outcomes confirmed the interaction between MSP1P-19 and band3-L5 (Fig. 4*D*).

The specific binding of MSP1P-19 to band3-L5 was demonstrated by competitive binding assays where interactions between GST-tagged band3-L5 and MSP1P-19 were inhibited by increasing the concentration of band3-P5 peptide (Fig. 4, *E* and *F*); the binding specificity was also proved by the antibody inhibition assays where the binding of band3-L5 to MSP1P-19 was blocked by anti-band3-P5 antibody in a dilution-dependent manner (Fig. 4, *G* and *H*).

### Reticulocyte receptor CD71 interacts specifically with MSP1P-19

**Figure 5:**
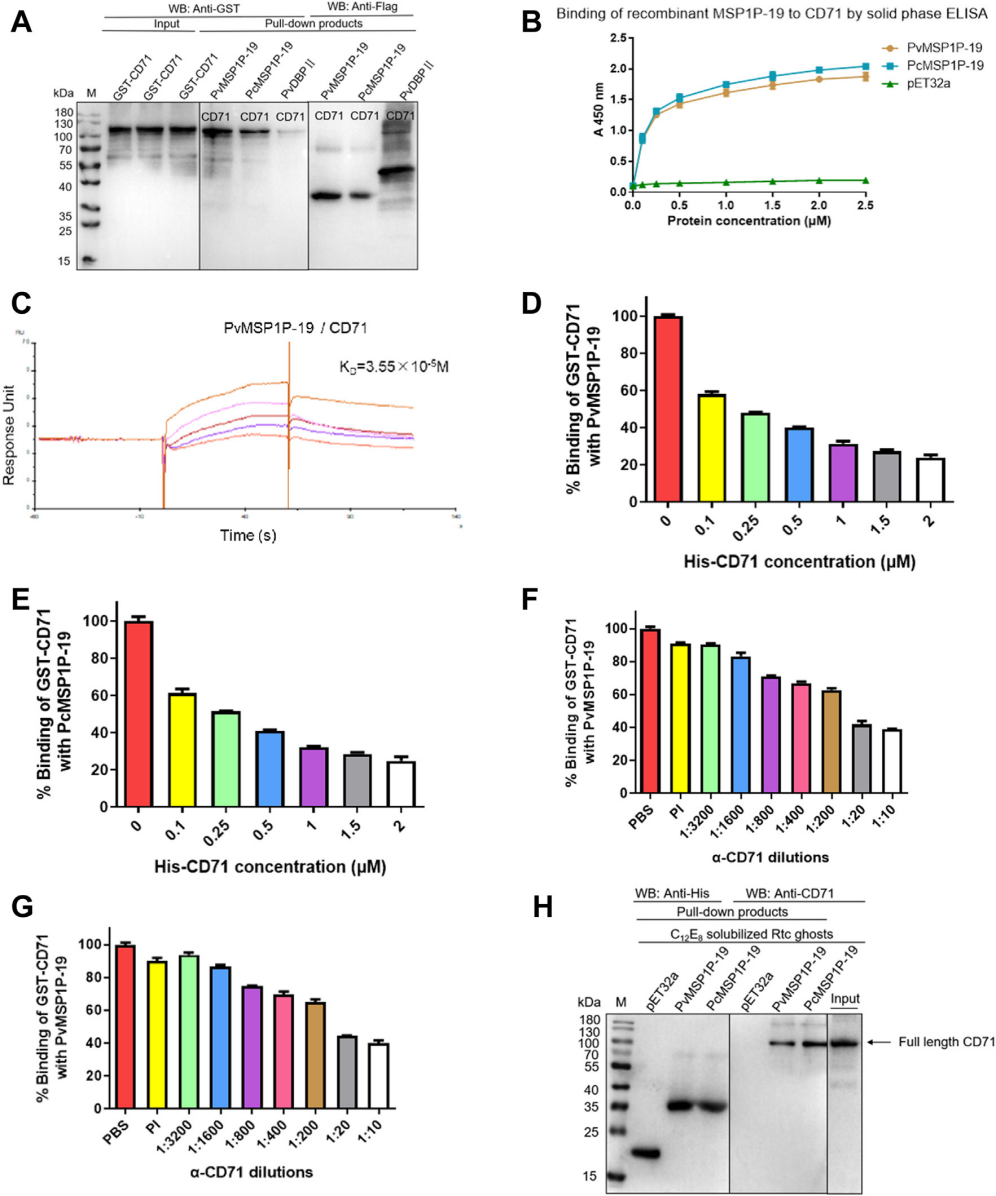
**Binding of MSP1P-19 to CD71.***A*, Flag-pull-down assays. The binding of MSP1P-19 to GST-CD71-ECD was confirmed by incubating GST-CD71-ECD proteins with immobilized Flag-MSP1P-19 or PvDBPII. After washing, the eluted complexes were detected using anti-GST and anti-Flag antibodies in Western blot analysis. *B*, solid-phase ELISA. Increasing concentrations of His-MSP1P-19 proteins and Trx-His tag protein (negative control) (0–2.5 μM) were incubated with ELISA plates coated with GST-CD71-ECD followed by detection using mouse anti-His antibody and goat anti-mouse antibody. *C*, SPR analysis of interactions between PvMSP1P-19 and CD71. Different concentrations of recombinant PvMSP1P-19 (6.25, 12.5, 25, and 50 μM) were injected over the surface of a CM5 chip immobilized CD71 at the flow rate of 30 μl/min. *D* and *E*, specificity of binding of MSP1P-19 to CD71 by competition assay. Different concentrations of His-CD71-ECD proteins (0–2 μM) were incubated with wells of an ELISA plate coated with PvMSP1P-19 (*D*) or PcMSP1P-19 (*E*) protein. After washing, the GST-tagged CD71-ECD (2 μM) was added to the wells followed by detection using anti-GST antibodies. With the binding of the group without adding His-CD71-ECD performed as percentage control, the mean value is plotted with S.D in three independent experiments*. F* and *G*, antibody inhibition assays. Specificity of binding of MSP1P-19 to CD71 was proved by incubating different dilutions of antibodies against CD71-ECD with immobilized GST-tagged CD71-ECD. A fixed concentration (2 μM) of Flag-tagged PvMSP1P-19 (*F*) or PcMSP1P-19 (*G*) protein was added to the wells after washing, followed by detection using Flag monoclonal antibody. The mean value of three independent experiments is plotted with S.D. *H*, interactions of MSP1P-19 to native CD71 confirmed by His-pull-down assays with Trx-His tag protein served as a negative control. CD71-ECD, extracellular domain of CD71; His, histidine; PvMSP1P, *P. vivax* merozoite surface protein 1 paralog; PvMSP1P-19, 19-kDa C-terminal region of PvMSP1P; SPR, surface plasmon resonance; Trx, thioredoxin.

Series of experiments were performed to confirm the specific interactions between MSP1P-19 and CD71. The extracellular domain of CD71 (CD71-ECD) was PCR amplified with a C-terminal HA tag and cloned into the pGEX-6P-1 vector for the expression of GST-HA-tagged proteins. His-pull-down assays revealed that MSP1P-19 interacted with CD71 (Fig. 5*A*). Using Trx-His-tagged protein as a negative control, this was further supported by the results of dose-dependent ELISA assays (Fig. 5*B*). Surface plasmon resonance (SPR) assays also confirmed that CD71 bound to MSP1P-19, with an equilibrium dissociation constant (K_D_) value of 3.55 × 10^−5^ M (Fig. 5*C*), whereas PvDBPII, which served as a control, did not bind to CD71 (Fig. S3).The binding specificity was confirmed using competitive binding assays where the interactions between GST-CD71-ECD and MSP1P-19 were increasingly inhibited at higher concentrations of His-CD71-ECD (Fig. 5, *D* and *E*); the specificity was also demonstrated in antibody inhibition assays where the binding was blocked by anti-CD71 polyclonal antibody in a dilution-dependent manner (Fig. 5, *F* and *G*).

To determine whether MSP1P-19 proteins interact with native CD71 protein, we performed the His-pull-down assays. The human reticulocyte membrane proteins were incubated with either a Trx-His-tagged protein (negative control) or MSP1P-19 protein which was immobilized on beads. Results showed that the native CD71 protein was pulled down by PvMSP1P-19 or PcMSP1P-19 protein immobilized to beads and recognized by mouse anti-CD71-ECD antibody (Fig. 5*H*). These findings confirmed that CD71 is the specific binding receptor of reticulocytes, which binds to MSP1P-19.

### Band3 is a specific chymotrypsin-sensitive receptor for MSP1P-19

**Figure 6:**
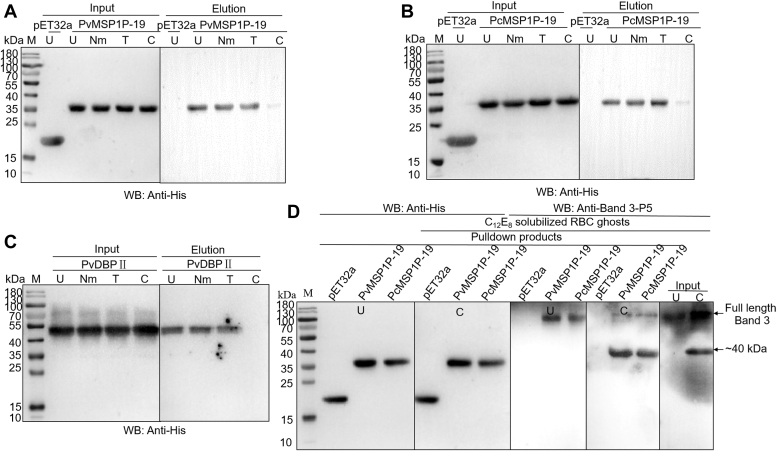
**The binding of band3 to MSP1P-19 is chymotrypsin-sensitive.***A*–*C*, His-tagged PvDBPII (*A*), PvMSP1P-19 (*B*), and PcMSP1P-19 (*C*) were respectively incubated with erythrocytes which were untreated (U), neuraminidase-treated (Nm), trypsin-treated (T), or chymotrypsin-treated (C). The eluted proteins were detected by mouse anti-His and goat anti-mouse antibodies in Western blots assays. The results showed that chymotrypsin treatment abolished the binding of PvDBPII or MSP1P-19 to band3. *D*, with Trx-His tag protein as a negative control, untreated (U) or chymotrypsin-treated (C) erythrocyte ghosts were incubated with His-tagged PvMSP1P-19 or PcMSP1P-19 protein bound to beads. The eluted proteins were detected by mouse anti-band3-P5 sera in Western blots assays. In chymotrypsin-treated group, ∼40 kDa fragment was specially detected.

The specificity of the erythrocyte receptor of MSP1P-19 was confirmed by enzyme-treated erythrocyte-binding assays where human erythrocytes were treated with neuraminidase (Nm), trypsin (T), or chymotrypsin (C), respectively. The pretreatment of Nm or T slightly reduced the binding affinity of PvDBPII or MSP1P-19 to erythrocytes according to the results, whereas pretreatment of C completely blocked the interaction between PvDBPII or MSP1P-19 and erythrocytes (Fig. 6, *A*–*C*). The above results confirmed previous reports of a C-sensitive interaction between MSP1P-19 and erythrocytes (19). Since band3 was previously identified as a C-sensitive erythrocyte receptor (24, 29), we wanted to find out whether it exhibits similar properties in interactions with MSP1P-19. His-pull-down assays were therefore conducted by incubating untreated (negative control) or C-treated erythrocyte ghosts with His-tagged MSP1P-19 proteins bound to beads. The results showed that anti-band3-P5 sera could recognize the full-length of band3 and another ∼40 kDa band. And, the ∼40 kDa fragment was pulled down by MSP1P-19 immobilized on beads in C-treated group (Fig. 6*D*). To sum up, the results indicated that MSP1P-19 binds to erythrocytes *via* the ∼40 kDa fragment of band3 in a C-sensitive manner.

### Antibodies against MSP1P-19, band3-P5, and CD71-ECD inhibit the binding of MSP1P-19 to reticulocytes

**Figure 7:**
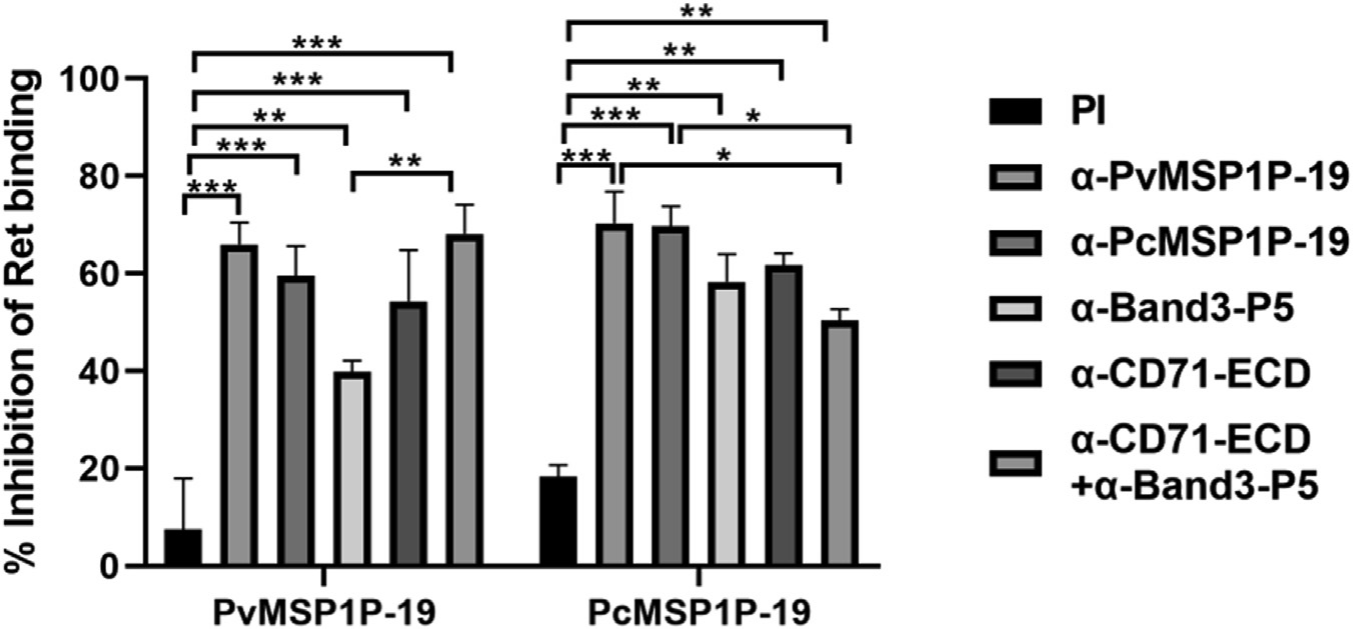
**Inhibition of the binding of MSP1P-19 to reticulocytes by anti-MSP1P-19, anti-band3-P5 or anti-CD71-ECD antibodies.** Inhibitory effect of antisera on binding of reticulocyte analyzed by flow cytometry. PvMSP1P-19 or PcMSP1P-19 protein was incubated with reticulocytes in the presence or absence of antibody. The binding capacity of MSP1P-19 to reticulocytes in the absence of antibody was normalized as a standard. Data are expressed as mean ± S.D in three independent experiments. (∗*p* < 0.05, ∗∗*p* < 0.01, ∗∗∗*p* < 0.001). CD71-ECD, extracellular domain of CD71; MSP1P surface protein 1 paralog; MSP1P-19, 19-kDa C-terminal region of MSP1P; PI, pre-immune mouse sera.

The *in vitro* reticulocyte-binding assay was carried out for further investigation of the inhibition capability of mouse antisera against MSP1P-19, band3-P5, or CD71-ECD on the binding of MSP1P-19 to reticulocytes. Recombinant PvMSP1P-19 and PcMSP1P-19 proteins were incubated with reticulocytes, and the ability of the antibodies to block the binding of recombinant MSP1P-19 was assessed by flow cytometry. The results showed that the binding of MSP1P-19 to reticulocytes was dramatically inhibited by antibodies against MSP1P-19, band3-P5, and CD71-ECD compared to negative control group (Fig. 7). In addition, in a HEK293T cell-based reticulocyte-binding assay, we found that binding of the MSP1P-19-expressing 293T cells to reticulocytes was significantly inhibited by antibodies against MSP1P-19, band3-P5, and CD71-ECD (Fig. S4).

### MSP1P-19 blocks *in vitro P. falciparum* invasion in a dose-dependent manner

**Figure 8:**
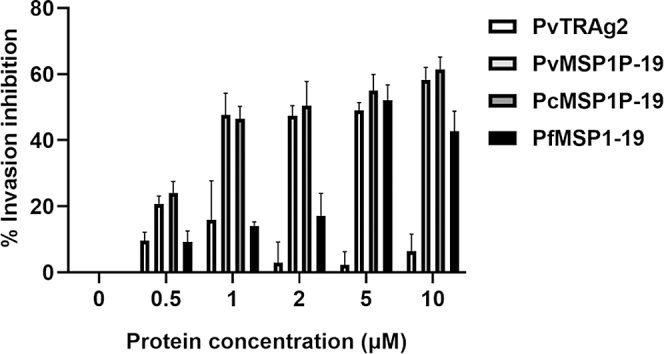
**MSP1P-19 inhibited the invasion ability of *P. falciparum*.** With PfMSP1-19 as a positive control and with PvTRAg2 as a negative control, different concentrations of PvMSP1P-19 or PcMSP1P-19 protein (0–10 μM) were incubated with synchronized parasites. Live cells were gated, and parasitemia was detected by flow cytometry 18 h later. Invasion ability of *P. falciparum* was significantly blocked by MSP1P-19 proteins in a dose-dependent manner. Data shown are the mean ± S.D for three independent experiments. PvMSP1P, *P. vivax* merozoite surface protein 1 paralog; PvMSP1P-19, 19-kDa C-terminal region of PvMSP1P.

To further elucidate the molecular mechanism of the interaction between MSP1P-19 and band3/CD71, the *in vitro* invasion assays were carried out. Synchronized parasite cultures were incubated with MSP1P-19 and PvTRAg2 proteins, and the infection rates were assessed using flow cytometry under the standard culture conditions of malaria (Fig. S5). Compared with nonerythrocyte-binding protein PvTRAg2 (30), the ability of *P. falciparum* for *in vitro* invasion was dramatically inhibited by MSP1P-19 proteins in a dose-dependent manner. At the dose of 10 μM, the invasion inhibition rates of *P. falciparum* in PvMSP1P-19 and PcMSP1P-19 groups were up to 58% and 61%, respectively (Fig. 8). The positive control PfMSP1-19 also showed inhibition of *P. falciparum* invasion.

## Discussion

The invasion of erythrocytes by *Plasmodium* is a complex process involving numerous host–parasite molecular interactions (31, 32). Identification of molecules involved in host–parasite interactions, as well as elucidation of the molecular mechanisms underlying these interactions, is critical for the production of new malarial drugs and vaccines. PvMSP1P-19 is a novel candidate for a malaria vaccine, and little information is available on the molecular mechanism of its interaction with reticulocytes. This study identified band3 and CD71 as reticulocyte receptors for PvMSP1P-19 and also showed that anti-band3-P5 and CD71-ECD antibodies inhibited the binding of MSP1P-19 to these receptors. Identifying PvMSP1P-19 reticulocyte receptors and elaborating the mechanism underlying this ligand–receptor interaction can improve our awareness of *P. vivax* invasion and aid in the production of malaria vaccines.

While *P. falciparum* can invade erythrocytes at all stages of development, *P. vivax* parasites have a strict preference for reticulocytes (23). On a *Plasmodium* invasion of host cells, the merozoites make contact and rapidly target erythrocytes. It is well understood that specific ligand–receptor interactions are required for *Plasmodium* merozoites to invade erythrocytes. However, since *P. vivax* parasites were unable to be cultured *in vitro* for a prolonged period, only a handful of specific receptors for *P. vivax* invasion of reticulocytes are known which include Duffy antigen, CD71, and CD98 (14, 16, 33). The *P. falciparum* erythrocyte-binding antigen 175 (EBA-175) binds to glycophorin A on the surface of erythrocyte in a sialic acid-dependent manner (34), and antibodies against its binding region can partially inhibit *P. falciparum* invasion (35). As a result, EBA-175 is regarded as an ideal blood-stage vaccine candidate for *P. falciparum* malaria (36, 37). A homolog to EBA-175 is the *P. falciparum* erythrocyte-binding antigen 140, which has been shown to bind to glycoprotein C on erythrocytes surface and reported to participate in the invasion pathway. Antibodies against erythrocyte-binding antigen 140 led to the reduction of merozoite invasion (38). PvMSP1P-19 has been proved to bind to human erythrocytes (39), independent of the Duffy antigen (19). In previous reports, PvMSP1P-19 elicited high levels of acquired immune responses in patients whose sera and specific antibodies inhibited the binding of PvMSP1P-19 to erythrocytes in a concentration-dependent manner (17, 19). Furthermore, anti-PvMSP1P-19 antibodies inhibited *P. vivax* invasion during short-term *in vitro* culture (19). This suggested that PvMSP1P-19 is essential in parasite invasion of erythrocytes (17, 40). In this study, band3 and CD71 were identified as reticulocyte membrane–protein receptors that bind to PvMSP1P-19 in enriched reticulocytes obtained from cord blood (Table 1). *Plasmodium* merozoites can invade erythrocytes through multiple pathways. As the most abundant erythrocyte membrane protein, band3 serves as the essential erythrocyte receptor for the invasion of several *Plasmodium* parasites (2, 24, 25, 41, 42, 43). Monoclonal antibodies against band3 were reported to block the invasion of *Plasmodium knowlesi* merozoites into rhesus erythrocytes (44). The three extracellular loop regions of band3 were expressed and purified based on LC-MS results. The experimental results confirmed that the key regions that bound specifically to PvMSP1P-19 were band3-L5 (Fig. 4). In order to determine the invasion pathway of MSP1P-19, further studies revealed that MSP1P-19 proteins were unable to bind to C-treated erythrocytes (Fig. 6), which was consistent with previous findings (19). While band3 was previously proved to be a C-sensitive receptor for *P. vivax* (2), we discovered that MSP1P-19 bound to erythrocytes *via* a 40 kDa fragment that was C sensitive (Fig. 6*D*).

Apart from band3, CD71 was found to have the maximum number of unique peptide segments (Table 1). CD71 is a transmembrane protein that binds to transferrin and participates in the transport of iron in cells (45). According to reports, the transformation of reticulocytes into mature erythrocytes was associated with the loss of several surface proteins, most notably complete loss of CD71 expression (46, 47). CD71 is made up of three domains: an N-terminal cytoplasmic domain, a transmembrane domain, and a C-terminal ECD containing a transferrin-binding site (45). CD71 is a receptor for the invasion of many pathogens (48, 49, 50). *P. vivax* can invade reticulocytes by interacting with the same CD71 receptor epitope as arenaviruses (14). Interaction of CD71 with *P. vivax* reticulocyte-binding protein 2b is reported to be essential in invasion of *P. vivax* parasites (14, 15). We confirmed the specific interactions of PvMSP1P-19 with band3-L5 and the full fragment of the CD71-ECD in this study. We will further need to segment the expression of the CD71-ECD based on its structure and function, as well as screen the key binding region of CD71 to PvMSP1P-19, to understand the molecular mechanism of PvMSP1P-19 binding to reticulocytes.

We also incubated HEK293T cells expressing MSP1P-19 with reticulocytes, and the results showed that the reticulocyte preference of MSP1P-19 was consistent with that of the *P. vivax* parasite (Fig. 2). In addition, the binding of MSP1P-19 proteins to reticulocytes was inhibited by antibodies against MSP1P-19, band3-P5, and CD71-ECD (Fig. 7). This suggested that merozoites attach to erythrocytes *via* band3 and CD71 during invasion. It should be noted that antibodies against MSP1P-19 inhibited the binding of MSP1P-19 to reticulocytes more than antibodies against CD71 and band3-P5 (Fig. 7). Since band3 was not associated with reticulocyte preference of *P. vivax* (14, 16, 51), we therefore concluded that band3 was likely to be involved in *plasmodium* invasion together with CD71 and that their interaction was essential for the invasion. At present, we cannot determine how band3 is involved in the process of CD71-mediated reticulocyte invasion, and the interdependence of band3 and CD71 receptor needs to be further explored in the future. Apical membrane antigen 1 (AMA1) is a blood-stage vaccine candidate, and AMA1 has similar antibody titers to the AMA1–rhoptry neck protein 2 complex, but the latter induces antibodies that provide more effective protection (52). The ability of antibodies induced by MSP1P-19 and band3 or CD71 complexes to block MSP1P-19–reticulocyte binding and the efficacy of protection can be investigated in the future. Furthermore, antibodies against PvMSP1P-19 or PcMSP1P-19 inhibited the binding of PvMSP1P-19 or PcMSP1P-19 protein to reticulocytes (Fig. 7). This suggested that PvMSP1P-19 and PcMSP1P-19 had similar antigenic determinant clusters and cross-reactivities, implying that *P. cynomolgi* parasites could be used to test the *in vitro* invasion inhibition ability of antibodies against PvMSP1P-19 and assess the potential of PvMSP1P-19 as a promising vaccine candidate.

The development of the vaccine relies on host–*Plasmodium* interactions. *Plasmodium* merozoites, particularly those of *P. falciparum* and *P. knowlesi*, have previously been shown to invade erythrocytes *via* multiple pathways (53, 54, 55, 56). The invasion ability of *Plasmodium* is enhanced by invasion redundancy. Band3 is an erythrocyte receptor for PfMSP1 invasion (24), and antibodies against band3 block *P. falciparum* invasion of human erythrocytes *in vitro* (42). Based on previous research, we used *P. falciparum* as a model to conduct *in vitro Plasmodium* invasion inhibition assays. Results showed that MSP1P-19 proteins could inhibit *P. falciparum* invasion in a concentration-dependent manner (Fig. 8). This also suggested that band3 was a common receptor for both *P. falciparum* and *P. vivax* erythrocyte invasions, and MSP1P-19 proteins competed with *P. falciparum* merozoites for the band3 receptor. A single *Plasmodium* ligand can recognize multiple erythrocyte receptors, and multiple *Plasmodium* ligands can recognize the same erythrocyte receptor. This phenomenon increases the invasion efficiency of parasites and also reflects the complexity of the invasion. This compelled us to screen for inhibitors of MSP1P-19 interactions with band3 and CD71 and test the invasion inhibition abilities using *in vitro P. cynomolgi* invasion inhibition assay.

Taken together, this study indicated that interactions of MSP1P-19 with band3 and CD71 were crucial for reticulocyte binding, and this information could provide insights into the production of novel antimalarial drugs and vaccines.

## Experimental procedures

### Ethics statement

The human studies covered in the manuscript adhere to the Declaration of Helsinki. Healthy blood samples were acquired from informed consenters. The Medical Ethics Committee and Animal Ethics Committee of Jiangnan University had respectively approved this study (JNU20210918IRB02) (Jn. no. 20200530b0301031).

### Preparation of recombinant MSP1P-19 proteins

**Supporting Figure S1:**
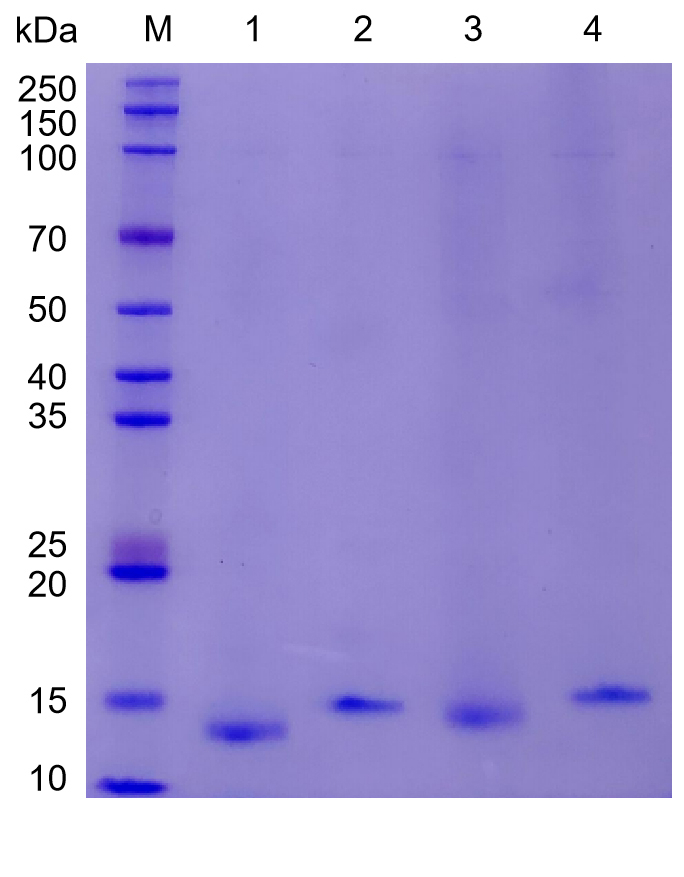


The full-length gene sequences of pvmsp1p and pcmsp1p (PlasmoDB ID: PVX_099975 and PCYB_073760) were acquired from PlasmoDB (http://plasmodb.org/plasmo/). The codon-optimized DNA sequences encoding *pvmsp1p-19* (aa 1749–1834) and *pcmsp1p-19* (aa 1739–1824) both with a C-terminal Flag tag were synthesized by Talen Biotech and cloned into pET32a and pEGFP-HSVgD1 vectors. The *E. coli* BL21 (DE3) and SHuffle T7 *E. coli* transfected with pET32a-MSP1P-19 plasmids were cultured in the Luria–Bertani medium and were induced with 0.5 mM isopropyl β-d-1-thiogalactopyranoside at the absorbance at 600 nm of 0.4 to 0.6 and were cultured at 16 °C for 20 h. After centrifugation, the soluble MSP1P-19 proteins were obtained and purified by Talen Biotech (57). Expression of soluble recombinant proteins was verified by SDS-PAGE and Western blot analysis using anti-His antibody. SDS-PAGE and circular dichroism experiments of MSP1P-19 with Trx removed were used to verify protein folding (Fig. S1 and Table S3).

### SDS-PAGE and Western blot analysis

The purified recombinant proteins were mixed with 5 × SDS reducing loading buffer and boiled at 100 °C for 10 min. The supernatant was then separated on 10% SDS-PAGE and stained with Coomassie Brilliant Blue.

In Western blot analysis, the recombinant proteins were transferred to the polyvinylidene difluoride membranes by electrophoresis and blocked using 5% skimmed milk diluted in PBS containing 0.2% Tween 20 (PBST) for 2 h at room temperature (RT). After washing, the polyvinylidene difluoride membranes were incubated with primary antibodies at 4 °C for 12 h followed by incubation using secondary antibodies at RT for 2 h. Immunoblots were visualized by enhanced chemiluminescence kit (New cell & Molecular Biotech), and the data were measured using ChemiDoc MP imaging system (Bio-Rad).

### Erythrocyte rosette assay

**Supporting Figure S2:**
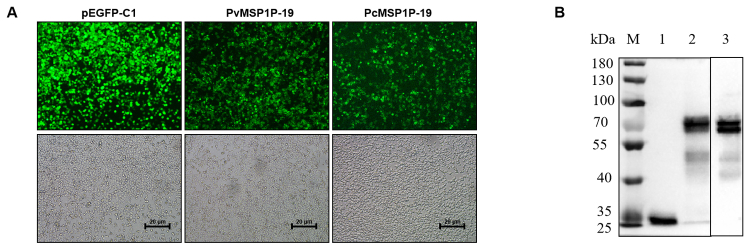


**Supporting Figure S3:**
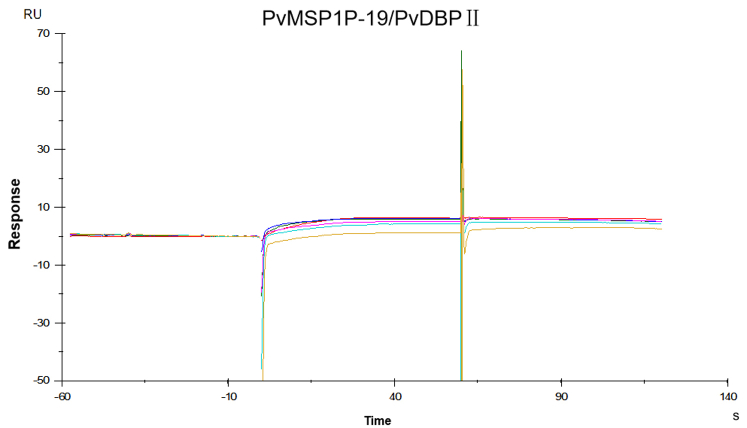


**Supporting Figure S4:**
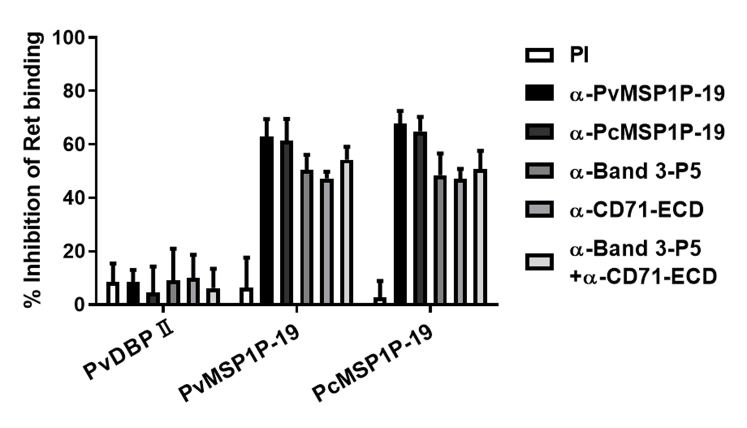


As described earlier (17), the *pvmsp1p and pcmsp1p* genes were PCR amplified using primers listed in Table S4 from pUC57-PvMSP1P-19 or pUC57-PcMSP1P-19 vectors and cloned into the pEGFP-HSVgD1 vectors. Fusion proteins of MSP1P-19 with the N-terminal signal sequence and C-terminal transmembrane region of herpes simplex virus glycoprotein D were obtained (58). The plasmids were purified and transfected into HEK293T cells with Lipofectamine LTX & PLUS reagent (Invitrogen) followed by incubation for 26 h (5% CO2, 37 °C). Fluorescence through the cell membrane was observed under an inverted fluorescence microscope. Fluorescence expressed across the cell membrane was seen compared to the control (pEGFP-C1, a plasmid that expresses green fluorescence within the cell) (Fig. S2*A*). Membrane proteins of transfected 293T cells were then collected, and the expression of MSP1P-19 proteins was detected by Western blot analysis using anti-GFP antibody (Fig. S2). Erythrocytes were added to the plates and incubated for 2 h. After washing, the number of rosettes (more than half of the surface of each transfected HEK293T cell was covered by erythrocytes) was counted under the bright field of a fluorescence microscopy in ten fields at 200× magnification. The relative binding capacities were normalized to the binding ability of PvDBPII (100% HEK293T cell transfection efficiency) with 0.8% reticulocyte.

For reticulocyte-binding assay, reticulocytes at 50% hematocrit were preincubated with diluted pre-immune mouse sera or antibodies against MSP1P-19, band3-P5, and CD71-ECD (1:100) followed by incubation with transfected HEK293T cells expressing PvDBPII, PvMSP1P-19, and PcMSP1P-19. The binding assays were then performed as described above.

### Co-affinity purification and LC-MS analysis

The reticulocyte membrane was obtained as described by Dodge *et al.* (59). In brief, after being washed for three times, the packed reticulocytes were incubated with 40 times the volume of Tris-HCl buffer. The mixture was then transferred into a magnetic stirrer for 20 min followed by centrifugation (10,000 rpm, 4 °C, 20 min). The above steps were repeated until erythrocyte ghosts turned into white pellets. Finally, the pellets were added to two times the volume of 1% C_12_E_8_ solubilization buffer (pH 8.0) and placed on ice for 20 min. The soluble reticulocyte membrane proteins were collected after centrifugation. The pre-equilibrated HisPur Ni-NTA Resin (Thermo Fisher Scientific) was respectively incubated with Trx-His tag protein and Trx-His-PvMSP1P-19 proteins (70 μg) at 4 °C for 3 h. After washing, the resin was incubated with reticulocyte membrane proteins (300 μg) at 4 °C for 12 h. The unbound proteins were eliminated by washing the resin, and combined compounds were then eluted with elution buffer (200 μl). After being mixed with 5 × SDS reducing loading buffer, the eluted proteins along with the purified His-tagged proteins were boiled and loaded onto SDS-PAGE, followed by staining with a rapid silver staining kit (Beyotime Biotechnology).

Compared with purified proteins group, differential strips and blank strips (blank control) were cut off followed by digestion as previously described (60, 61). LC-MS analysis were performed using a Q-Exactive mass spectrometer (Thermo Fisher Scientific) and an Easy-nLC chromatography system (Thermo Fisher Scientific). Proteome Discoverer 1.4 software (Thermo Fisher Scientific) was used to generate peak lists and perform database searches to obtain identification results.

### Preparation of band3 and CD71 proteins

The genes encoding three extracellular loop regions of band3 and CD71-ECD were amplified from the cDNA (prepared from human reticulocyte mRNA) and cloned into *BamH*1/*Xho*1 sites of GST-pGEX-6P-1 and His-pET28a vectors. To obtain recombinant HA-tagged band3 and CD71 proteins, HA tag nucleotide sequence was added into their reverse primers (Table S4). The aa sequences of band3 fragments were listed in Table S5. Detailed procedures of preparation of band3 and CD71 proteins were the same as previously mentioned.

### Acquisition of antisera against MSP1P-19, band3-P5, and CD71

To obtain anti-MSP1P-19 antisera, five female BALB/c mice (6–8 weeks old) were immunized with MSP1P-19 containing Freund’s complete adjuvant (Sigma) by intraperitoneal injection followed by two intraperitoneal injections of MSP1P-19 containing Freund’s incomplete adjuvant (Sigma) at an interval of 3 weeks. One week after the final booster, mouse anti-MSP1P-19 antisera were collected. Band3-P5 peptide was synthesized to prevent immune response against GST (Table S6).

### Pull-down assays

Flag-pull-down assays were performed for confirmation of the specific binding of MSP1P-19 to band3 or CD71-ECD and find out the core binding region. MSP1P-19 or PvDBPII protein (20 μg) was incubated with anti-Flag M2 magnetic beads (20 μl) at 4 °C for 3 h. A magnetic separator was used to collect beads followed by washing for three times with TBS. Recombinant GST-tagged extracellular loops of band3 and CD71 proteins were then added to beads and incubated at 4 °C for 12 h. After washing, the beads were mixed with 1 × SDS reducing loading buffer (50 μl) followed by boiling. The supernatant was then collected, and Western blot assays were carried out.

His-pull-down assays were then carried out to confirm the binding of MSP1P-19 to native CD71 protein, where reticulocyte ghost lysates were incubated with PvMSP1P-19 or PcMSP1P-19 immobilized on magnetic beads. After washing, Western blot assays were carried out as previously mentioned.

### ELISA

ELISA assays were performed for confirmation of the binding of MSP1P-19 to band3 and selection of their core binding region. Each well of a 96-well microtiter plate was coated with PvMSP1P-19 or PcMSP1P-19 protein (100 ng) which dissolved in carbonate buffer for 12 h at 4 °C. Then, the plates were washed with PBST for three times and blocked with 0.5% BSA dissolved in PBST for 2 h at RT. After washing for three times, the plates were incubated with different concentrations of GST-tagged proteins for 2 h at RT. The plates were then incubated with the rabbit anti-GST antibody for 2 h. After being incubated with the goat anti-rabbit IgG antibody, the plates were then incubated with 1 × TMB and stored in dark at RT for 3 min. Finally, the reaction was stopped using 2 M H_2_SO_4_ (50 μl per well), and the absorbance was measured at 450 nm.

To eliminating the effects of GST, Trx-His tag protein and recombinant MSP1P-19 proteins were incubated with band3-L5 coated on microtiter plates in ELISA assays. To confirm the interactions between MSP1P-19 and CD71, increasing concentrations of Trx-His tag protein and His-MSP1P-19 proteins were incubated with ELISA plates coated with GST-CD71-ECD. The bound proteins were detected as described earlier.

In competitive binding assays, ELISA assays were conducted as described earlier. Briefly, increasing concentrations of untagged band3-P5 or His-CD71-ECD proteins were incubated with 96-well plates coated with PvMSP1P-19 or PcMSP1P-19 (50 ng). After washing, the plates were then incubated with GST-tagged band3-L5 or CD71-ECD (2 μM) followed by detection with GST monoclonal antibody, as described above.

For antibody inhibition assays, the 96-well microtiter plates coated with GST-tagged band3-L5 or CD71-ECD proteins were incubated with different dilutions of mouse anti-band3-L5 or anti-CD71-ECD antibodies. After washing, the plates were incubated with Flag-tagged PvMSP1P-19 or PcMSP1P-19 protein (2 μM) followed by detection with Flag monoclonal antibody, as described earlier.

### SPR analysis

To prove the binding affinity of PvMSP1P-19 to CD71-ECD, SPR assays were carried out using a BIAcore S200 instrument (GE Healthcare). The CM5 sensor chips were activated by flowing the mixture of 1:1 1-ethyl-3-(3-dimethylamino) propylcarbodiimide hydrochloride and N-hydroxysuccinimide. Recombinant CD71-ECD dissolved in 10 mM NaAC (pH 4.5) was bound to flow cell 2 at a flow rate of 10 μl/min. Different concentrations of analyte (PvMSP1P-19) were allowed to flow through immobilized CD71-ECD at a flow rate of 30 μl/min. The 10 mM glycine-HCl (pH 2.0) was used for regeneration of the CM5 sensor chip. BIAevaluation software (GE Healthcare) was used to analyze concentrations of analyte and binding responses at steady state followed by fitting the corrected response data to a 1:1 Langmuir binding model. The apparent equilibrium dissociation constant (K_D_) was calculated by the equation K_d_ = k_d_/k_a_, in which k_d_ and k_a_ respectively represent the dissociation and association rate constants.

### Enzymatic treatments to erythrocytes and binding assays of MSP1P-19 to erythrocyte

The erythrocytes were treated as previously published (24, 62). Enriched erythrocytes (250 μl) were respectively incubated with Nm (66.7 mU/ml), T (1 mg/ml), and C (1 mg/ml) at 37 °C for 1 h, followed by adding T inhibitor (0.5 mg/ml) and incubation at 37 °C for 10 min. The erythrocytes were then collected for binding assays after washing with incomplete RPMI 1640 for three times.

For binding assays, the enzyme-treated enriched erythrocytes along with the untreated erythrocytes were incubated with Trx-His tag protein, His-tagged MSP1P-19, or PvDBPⅡ protein at 37 °C for 1 h followed by centrifugation using silicon oil. The pellets were then collected and washed with PBS. After incubation with 0.5 M NaCl (50 μl), the supernatant was collected for Western blot assays.

### Flow cytometry-based reticulocyte-binding assay

The reticulocytes were prepared as described by Gruszczyk *et al.* (14). Briefly, enriched reticulocytes resuspended in PBS in the presence or absence of antibodies were incubated with recombinant MSP1P-19 proteins for 1 h at RT. After being washed for three times, the binding complexes were incubated with anti-His tag antibody (BioLegend) for 20 min on ice, shielded from light. After washing, thiazole orange (Solarbio) was added and incubated for 20 min. The reticulocytes were then collected and resuspended in PBS for further analysis on Accuri C6 plus flow cytometer (BD Biosciences). One hundred thousand events were collected and analyzed by FlowJo software.

### *P. falciparum* culture and invasion inhibition assays

*P. falciparum* 3D7 parasites were cultured in complete RPMI 1640 medium containing 2 g/L NaHCO_3_, 5 g/L AlbuMAX II (Sigma), and 50 mg/L hypoxanthine II (Sigma) using O^+^ human erythrocytes (4% hematocrit) in a 37 °C humidified incubator (5% O_2_, 5% CO_2_, and 90% N_2_). Parasite cultures were synchronized by treatments of sorbitol (63).

The synchronized parasites (1.5% parasitemia) at late trophozoite or early schizont stage were seeded in 96-well plates with a final volume of 100 μl per well followed by incubation with different concentrations of PvMSP1P-19 or PcMSP1P-19 protein (0–10 μm). PfMSP1-19 protein served as a positive control, while PvTRAg2 protein served as a negative control (30). After 18 h of incubation, the infected erythrocytes were fixed and then stained with SYBR green (Invitrogen). One hundred thousand total events were acquired per sample with the BD Accuri C6 plus flow cytometer, and the data were calculated with FlowJo software.

### Statistical analysis

All data were analyzed by GraphPad Prism software and Microsoft Excel 2016. Student’s *t* test was used to compare the differences between the groups to determine their statistical significance. Differences were statistically significant at *p* <0.05. One-way ANOVA and SNK-q test were used for the invasion inhibition assay.

## Data availability

All the data described in the manuscript are contained within the main manuscript or the Supporting information.

## Supporting information

This article contains supporting information (17, 28, 58)

## Conflict of interest

The authors declare no conflicts of interest with the contents of this article.

## Figures and Tables

**Supporting Figure S5:**