# Supplementary material for: The Plasmodium vivax MSP1P-19 is involved in binding of reticulocytes through interactions with the membrane proteins band3 and CD71
Source: J Biol Chem. 2024 Apr 16;300(5):107285. doi: 10.1016/j.jbc.2024.107285 (PMC11107369; doi:10.1016/j.jbc.2024.107285)
Supplement: Supporting Figure Legends [file mmc1.docx]

**Figure S1. SDS-PAGE gel of recombinant MSP1P-19.** Lane M, molecular weight marker; Lanes 1 and 3, purified PvMSP1P-19 (1) and PcMSP1P-19 (3) recombinant proteins in reducing condition; Lanes 2 and 4, purified PvMSP1P-19 (2) and PcMSP1P-19 (4) recombinant proteins in non-reducing condition.

**Figure S2. Confirmation of surface expression of MSP1P-19.** (A) Expression of GFP-MSP1P-19 on the surface of HEK293T cells observed under fluorescence microscopy. (B) Expression of GFP-MSP1P-19 on the surface of HEK293T cells analyzed by western blot. Lane M, molecular weight marker; Lane 1, pEGFP-C1; Lane 2, pEGFP-HSVgD1-PvMSP1P-19; Lane 3, pEGFP-HSVgD1-PcMSP1P-19.

**Figure S3. SPR analysis of interactions between PvMSP1P-19 and PvDBPⅡ.** Different concentrations of recombinant PvDBPⅡ (0, 0.625, 1.25, 2.5, 5, 10, 20 mM) were injected over the surface of a CM5 chip immobilized CD71 at the flow rate of 30 μl/min.

**Figure S4. Inhibition of the binding of MSP1P-19 to reticulocytes by anti-MSP1P-19, anti-band3-P5 or anti-CD71-ECD antibodies.** reticulocyte binding assay based on the HEK293T cell.

Reticulocytes were incubated with pre-immune (PI) mouse sera or mouse sera against MSP1P-19, band3-P5 and CD71-ECD followed by incubation with transfected HEK293T cells expressing PvDBPII, PvMSP1P-19 and PcMSP1P-19. The binding capacity of PvDBPII at 100% transfection efficiency was normalized as a standard. Binding of MSP1P-19 to reticulocytes was inhibited by mouse sera. Data are expressed as mean ± S.D in three independent experiments. (**P* < 0.05, ***P* < 0.01, ****P* < 0.001).

**Figure S5.** **Representative dot plots showing the rate of infection.** Panel a, unstained erythrocytes; panel b, uninfected erythrocytes; panel c, infected erythrocytes; panel d, infected erythrocytes treated with PBS; panel e, infected erythrocytes treated with PvMSP1P-19; panel f, infected erythrocytes treated with PcMSP1P-19; panel g, infected erythrocytes treated with PfMSP1-19; panel h, infected erythrocytes treated with PvTRAg2.
