# Supplementary material for: The Plasmodium vivax MSP1P-19 is involved in binding of reticulocytes through interactions with the membrane proteins band3 and CD71
Source: J Biol Chem. 2024 Apr 16;300(5):107285. doi: 10.1016/j.jbc.2024.107285 (PMC11107369; doi:10.1016/j.jbc.2024.107285)
Supplement: Supporting Tables S1–S6 [file mmc2.doc]

**Tables S1 to S6**

**Table S1. Identified peptide sequences of identified reticulocyte membrane proteins by LC-MS.**

| **No.** | **Protein** | **UniProt accession no.** | **No. of unique peptides** | **Peptide sequences** |
| --- | --- | --- | --- | --- |
| 1 | Anion transport protein ( Band 3) | P48751 | 24 | VLLPLIFR, ADFLEQPVLGFVR, FIFEDQIRPQDR, LQEAAELEAVELPVPIR, GTVLLDLQETSLAGVANQLLDR, HSHAGELEALGGVKPAVLTR, IPPDSEATLVLVGR, GLDLNGGPDDPLQQTGQLFGGLVR, ASTPGAAAQIQEVK, ATFDEEEGRDEYDEVAMPV,  FLFVLLGPEAPHIDYTQLGR, SVTHANALTVMGK, ATFDEEEGR, ILLLFKPPK, ALLSLVPVQ, IDAYMAQSR, RYQSSPAKPDSSFYK, SGDPSQPLLPQHSSLETQLFCEQGDGGTEGHSPSGILEK, NVELQCLDADDAK, ATFDEEEGRDEYDEVAMPV, YHPDVPYVK, GWVIHPLGLR, LSVPDGFK, YQSSPAKPDSSFYK |
| 2 | Transferrin receptor protein1 (CD71) | P02786 | 13 | SAFSNLFGGEPLSYTR, LDSTDFTGTIK, DSAQNSVIIVDK, LLNENSYVPR, VEYHFLSPYVSPK, GFVEPDHYVVVGAQR, LTHDVELNLDYER, LTTDFGNAEK, VANAESLNAIGVLIYMDQTK, HVFWGSGSHTLPALLENLK, AVLGTSNFK, LTVSNVLK, LVHANFGTK |
| 3 | Ankyrin -1 | P16157 | 9 | TAEAVNFATLLYK, LDQVVESPAIPR, TAAVLLQNDPNPDVLSK, LLGNQATFSPIVTVEPR, QQGQEEQVQEAK, LSTPPPLAEEEGLASR, LCQDYDTIGPEGGSLK, IIALGPTGAQFLSPVIVEIPHFASHGR, EGHVETVLALLEK |
| 4 | Solute carrier family 2, facilitated glucose transporter member 1 | P11166 | 2 | TFDEIASGFR, VTILELFR |
| 5 | 55 kDa erythrocyte membrane protein | Q00013 | 2 | FATGDIIQIINK, IAILDIEPQTLK |
| 6 | protein band 4.2 | P16452 | 1 | VALTAQTGEQPSK |

**Table S2. Codon-optimized sequences used in this study.**

| Name | Codon-optimized sequences |
| --- | --- |
| *pvmsp1p-19* | GACCGTGTTGAGAAGAATTGCCGTAACCGTAAGTGTCCGCTTAACTCATTTTGCTTTATTCAAACGATCAATGAAGAATGCCTGTGCTTGTTGAACTACAGCATGGTGGGTGAGAAATGCATTCTTAACGAGCAGAACTCATGCGCTGTCAAGAACGGTGGGTGCGACCTGAAAGCAACCTGTGAATTAAAGAAGAATCGCGTGAACTGCATCTGTCCCAAGGGGACAAAACCCATGCACGAGGGAGTTGTTTGCAGC |
| *pcmsp1p-19* | GAGAGTGTGGAGAAGGATTGTCGTAACCGCAAATGTCCAAGCAATAGTTTTTGCTTCATTCAGACATTCAATGAGAACTGCTTATGCTTCCTTAACTATAATATGGTAGGTGAAAAATGTATTTTCAATGAGCATAATACCTGTGATGTTAAAAATGGCGGATGTGATTCTAAGGCGACTTGCGTTATGAAGAAAAATCGCGTAATTTGCGTGTGCCCAAAAGGAACTAAACCCATTTACGAAGGTGTGGTATGTAAC |

**Table S3. Circular dichroism results**

|  | α-helix | β-sheet | β-turn | Random |
| --- | --- | --- | --- | --- |
| PvMSP1P-19 | 9.2% | 46% | 33.9% | 10.9% |
| PcMSP19-19 | 6.8% | 44% | 30.1% | 19.1% |
| CD71-ECD | 38.6% | 20.5% | 16.5% | 24.3% |

**Table S4. Primer sequences of *TFRC(CD71)* and *SLC4A1 (Band 3)* for recombinant protein expression and primer sequences of *pvmsp1p-19* and *pcmsp1p-19* genes for HEK293T cell surface expression.**

| Primer | Sequences (5'→3')^a^ |
| --- | --- |
| pET32a-PvMSP1P-19-F | gctgatatcGGATCCGACCGTGTTGAGAAGAATTGC |
| pET32a-PvMSP1P-19-R | gtggtggtgCTCGAGCTTATCGTCGTCATCCTTGTAATCGCTGCAAACAACTCCCTC |
| pET32a-PcMSP1P-19-F | gctgatatcGGATCCGAGAGTGTGGAGAAGGATTGTC |
| pET32a-PcMSP1P-19-R | gtggtggtgCTCGAGCTTATCGTCGTCATCCTTGTAATCGTTACATACCACACCTTCGTAAA |
| pEGFP-HSVgD1-PvMSP1P-19-F | ggtcctggacGAATTCGACCGTGTTGAGAAGAATTGC |
| pEGFP-HSVgD1-PvMSP1P-19-R | gtgtatggggccttGGGCCCCTTATCGTCGTCATCCTTGTAATCGCTGCAAACAACTCCCTC |
| pEGFP-HSVgD1-PcMSP1P-19-F | ggtcctggacGAATTCGAGAGTGTGGAGAAGGATTGTC |
| pEGFP-HSVgD1-PcMSP1P-19-R | gtgtatggggccttGGGCCCCTTATCGTCGTCATCCTTGTAATCGTTACATACCACACCTTCGTAAA |
| pGEX-6P-1-CD71-ECD-F | gggcccctgGGATAATGTAAAGGGGTAGAACCAAAA |
| pGEX-6P-1-CD71-ECD-R | atgcggccgCTCGAGAGCGTAATCTGGAACATCGTATGGGTAAAACTCATTGTCAATGTCCCA |
| pET28a-CD71-ECD-F | atgggtcgcGGATCCTGTAAAGGGGTAGAACCAAAA |
| pET28a-CD71-ECD-R | gtggtggtgCTCGAGAGCGTAATCTGGAACATCGTATGGGTAAAACTCATTGTCAATGTCCCA |
| pGEX-6P-1-Band 3-L4-F | gggcccctgGGATCCTTCATTCAGGATACCTACACCC |
| pGEX-6P-1-Band 3-L4-R | atgcggccgCTCGAGAGCGTAATCTGGAACATCGTATGGGTACATCCAGATGGGAAACTCG |
| pGEX-6P-1-Band 3-L5-F | gggcccctgGGATCCCTCAGTGCCACCACCGT |
| pGEX-6P-1-Band 3-L5-R | atgcggccgCTCGAGAGCGTAATCTGGAACATCGTATGGGTAGCGGGACAGGATGGG |
| pGEX-6P-1-Band 3-L6-F | gggcccctgGGATCCGACCGCATCTTGCTTCTG |
| pGEX-6P-1-Band 3-L6-R | atgcggccgCTCGAGAGCGTAATCTGGAACATCGTATGGGTACAGGGAGGCCGGC |

^a^The vector sequences are lowercase, and the restriction sites are underlined. FLAG tag sequences and HA tag sequences are highlighted in gray.

**Table S5. Amino acid sequences of band 3 fragments expressed in this study.**

| Fragment | Amino acid sequences |
| --- | --- |
| L4 | FIQDTYTQKLSVPDGFKVSNSSARGWVIHPLGLRSEFPIWM |
| L5 | ALFGMPWLSATTVRSVTHANALTVMGKASTPGAAAQIQEVKEQRISGLLVAVLVGLSILMEPILSRIPLAVLFGIFLYMG |
| L6 | DRILLLFKPPKYHPDVPYVKRVKTWRMHLFTGIQIICLAVLWVVKSTPASL |

**Table S6. Amino acid sequence of synthetic peptides used in this study.**

| Peptide name | Amino acid sequence |
| --- | --- |
| Band 3-P5 | LSATTVRSVTHANALTVMGKASTPGAAAQIQEVKEQRISGLLVAVLVGLSILMEPILSR |
